# Portal dosimetry for pretreatment verification of IMRT plan: a comparison with 2D ion chamber array

**DOI:** 10.1120/jacmp.v11i4.3268

**Published:** 2010-08-19

**Authors:** Dayananda Shamurailatpam Sharma, Vaibav Mhatre, Malhotra Heigrujam, Kaustav Talapatra, Suman Mallik

**Affiliations:** ^1^ Department of Radiation Oncology Kokilaben Dhirubhai Ambani Hospital and Medical Research Institute Mumbai 400053 India

**Keywords:** portal dosimetry, IMRT, 2D detector array, gamma, QA

## Abstract

Portal dosimetry (PD) was performed for 181 fields from 14 IMRT plans of various clinical sites at gantry zero and source‐to‐detector distance (SDD) of 100 cm. PD was realized using aSi1000 electronic portal imaging device (EPID) and portal dose prediction (PDP) algorithm implemented in Eclipse treatment planning system (TPS). Agreement of PDP predicted and EPID measured photon fluence/dose distribution were evaluated using gamma (γ) index set at 3% at 3 mm distance to point agreement (DTA). Three gamma scaling parameters, maximum $\gamma {\left(\gamma \right)}_{\max}$, average $\gamma {\left(\gamma \right)}_{avg}$ and percentage of points with $\gamma \leq 1(\gamma_{\%}\leq 1)$ were estimated for each field. An independent measurement was carried out using MatriXX 2D ion chamber array with detector plane at 100 cm and $\gamma_{\max},\gamma_{avg}$ and $\gamma_{\%}\leq 1$ were estimated using OmniPro IMRT analyzing software. Effect of extended SDD and gantry rotation on portal dosimetry outcome was also investigated for another 45 IMRT fields. PDP predicted and EPID measured photon fluence agrees well with overall mean values of $\gamma_{\max},\gamma_{avg}$ and $\gamma_{\%}\leq 1$ 1 at 2.02, 0.24 and 99.43%, respectively. $\gamma {\left(\gamma \right)}_{\max}$ value was lower in 15 MV compared to 6 MV IMRT plan. Independent verification using MatriXX showed comparable overall mean values of $\gamma {\left(\gamma \right)}_{avg}$ and $\gamma_{\%}\leq 1$ at 0.25 and 99.80%. However, in all plans, MatriXX showed significantly lower $\gamma_{\max}(p<0.05)$ with an overall mean value of 1.35. In portal dosimetry, compared to gamma values at 100 cm SDD, $\gamma_{\max},\gamma_{avg}$ and $\gamma_{\%}\leq 1$ values improve from a mean of 0.16, 0.03 and 0.26 at 110 cm SDD to 0.35, 0.05 and 0.29 at 140 cm SDD. PD outcome was independent of gantry rotation. In conclusion, both MatriXX 2D ion chamber array and portal dosimetry showed comparable results and can be use as an alternative to each other for relative photon fluence verification.

PACS number (s): 87.55.D‐, 87.55.de, 87.55.kd,87.55.km,87.55.Qr,87.56Fc:

## I. INTRODUCTION

The complexity of intensity‐modulated radiotherapy (IMRT) demands thorough verification of planned radiation dose before treatment. Traditional methods of film dosimetry for pretreatment verification of patient‐specific IMRT dose distribution^(^ ^1^ ^–^ ^4^ ^)^ is gradually being replaced by two‐dimensional (2D) detector arrays due to their ease of use and immediate readout of the results. Dosimetric characteristics and clinical implementation of 2D detector arrays consisting of a large number of ionization chambers or diodes have been reported for pretreatment verification of IMRT plans.^(^ ^5^ ^–^ ^10^ ^)^ The pixel ionization chamber‐based 2D detector arrays, MatriXX (Scanditronix Wellhofer, Germany) is increasingly used for verification of IMRT plans due to its excellent dose and dose rate linearity, reproducibility, and insignificant energy and field size dependence for megavoltage photon beams.^(^ ^6^ ^–^ ^8^ ^,^ ^10^ ^)^

Van Herk^(^ ^11^ ^)^ proposed using electronic portal imaging device (EPID), available for patient set‐up verification for dose measurement. Dosimetry using EPIDs or portal dosimetry has received considerable attention recently after the development of new generation, high‐resolution amorphous silicon (aSi) flat‐panel detector and portal image to dose conversion software. The dosimetric characteristics and calibration procedures of various types of EPIDs, strategies to use EPIDs for dose verification, clinical approaches to EPID dosimetry and current clinical experiences were extensively reviewed and reported in a recent article by van Elmpt et al.^(^ ^12^ ^)^ The majority of the studies reporting EPID dosimetry have focused on dose response characteristic of detectors, and various methods and algorithms in use.^(^ ^11^ ^–^ ^17^ ^)^ However, a few have demonstrated the usefulness of portal dosimetry for pretreatment verification of IMRT, mostly using their in‐house developed portal image to dose conversion software.^(^ ^18^ ^–^ ^21^ ^)^ While limited data on the clinical application of these two emerging quality assurance (QA) methods are available separately, to our knowledge, no data are available comparing MatriXX results with that from portal dosimetry for the same IMRT fluence. This kind of comparative study is necessary to better understand the performance of each method, and gain the confidence of adopting a new method and establishing action levels for IMRT QA

Recently we commissioned aSi1000 EPID and portal dose prediction (PDP) algorithm in Eclipse (Varian Medical Systems, Palo Alto, CA) treatment planning system (TPS) for portal dosimetry. In this study, agreement of PDP algorithm predicted and EPID measured photon fluence/dose distribution were tested for clinically treated IMRT plans of various sites representing different intensity profiles. The portal dosimetry results were compared with an independent verification method employing MatriXX, 2D ion chamber array and OmniPro (Scanditronix Wellhofer, Germany) IMRT analyzing software. Effect of gantry rotation and extended source‐to‐detector distance (SDD) were also investigated on portal dosimetry outcome.

## II. MATERIALS AND METHODS

A high resolution aSi1000 EPID, available for patient set‐up verification on Trilogy linear accelerator (Varian Medical Systems, Palo Alto, CA) and portal dose prediction (PDP) software in Eclipse TPS (V8.5) was commissioned for portal dosimetry. The aSi1000 EPID has arrays of light sensitive amorphous‐Si photodiodes arranged in $40\times 30{\text{cm}}^{2}$ active detector area (1024×768 pixels, $0.039\times 0.034\;{cm}^{2}$ pixel pitch). Images are acquired using a frame averaging method with a fast frame grabbing rate of 30 frames per second (fps). The commissioning of portal dosimetry involves the configuration of PDP software and calibration of EPID response at different locations of the imager to the dose measured by a reference dosimeter following manufacturer recommended calibration protocol.^(^ ^22^ ^)^ Calibration was performed in special, integrated portal image acquisition mode for 6 and 15 MV X‐ray at 100 and 140 cm SDD and 400 MU/min, which is the dose rate used for IMRT delivery. Once calibrated, EPID signal is displayed in an arbitrary unit called calibration units (CU) and is related to monitor units (MU) and dose. The details of the calibration process are out of the scope of the present study. Portal dose prediction in Eclipse TPS uses dose reconstruction approach in the absence of patient/phantom per beam at the position of the EPID. After exposure on the calibrated EPID, the registered portal image of the photon fluence was processed for each field and compared against the corresponding predicted fluence using analysis software included in the system. The details of the of PD prediction algorithm available in Eclipse TPS and clinical commissioning has been described elsewhere.^(^ ^18^ ^)^

A total of 181 fields/subfields from 14 IMRT plans (four head and neck (H&N)), three cervix, six prostate and one brain) were selected for this retrospective study. All IMRT plans were created employing sliding window technique with 6 MV X‐rays in eight plans and 15 MV X‐rays in six plans. Dose computation was performed using Anisotropic Analytical Algorithm (AAA) at a grid size of $0.25\times 0.25\times 0.25\;{cm}^{3}$. Of the 14 IMRT plans, 2 plans had five fields, 11 plans had seven fields and 1 plan had nine fields. Intensity‐modulated fields with more than 14.5 cm width along MLC motion were automatically split into two subfields due to the physical limitation of the MLC. For every IMRT plan, a portal dose verification plan ($P_{\text{PD}}$) was created in Eclipse TPS using PDP algorithm. In $P_{\text{PD}}$, a photon fluence/dose distribution at the position of EPID kept at source‐to‐detector distance (SDD) of 100 cm was predicted per field/subfield at gantry zero degree without any phantom/patient. The same fluence was exposed on the EPID available on a Trilogy linear accelerator under the same geometry. The images acquired in integrated mode were processed to determine the photon fluence/dose distribution in CU at the EPID position. This processed fluence was then compared with the corresponding predicted fluence in Eclipse TPS using the analysis software included in the portal dosimetry system. Gamma (γ) evaluation method proposed by Low et al.^(^ ^23^ ^)^ was used for quantification of the results. Reference gamma index value was set at 3% dose agreement within 3 mm distance to agreement (DTA).^(^ ^24^ ^)^ For each fields/subfields, three scalar parameters, namely gamma maximum $\left(\gamma_{\max}\right)$, gamma average $(\gamma_{\mathrm{avg}}$), and percentage of the field area with all pixels having at most gamma value $1(\gamma_{\%}\leq 1)$ were evaluated to quantify the overall agreement between predicted and measured photon fluence/dose distribution.

An independent verification of TPS calculated and delivered dose distribution from the same IMRT plans were carried out for each field using an independent method comprising of MatriXX and OmniPro IMRT (V1.6) analyzing software. MatriXX is a 2D pixel ionization chamber array of 1020 detectors on a 32×32 Cartesian grid. The detector spacing is 0.76 cm, covering a total area of $23.6\times 23.6\;{cm}^{2}$. For every plan, IMRT verification plan ($P_{\text{MatriXX}}$) was also created in Eclipse TPS using 3D CT dataset of the MatriXX sandwich between two solid water slabs of 5 cm each. Fluence of every plan was projected separately at gantry zero and SDD of 100 cm and dose computation was performed using AAA at a grid size of $0.25\times 0.25\times 0.25\;{cm}^{3}$. Eclipse TPS calculated IMRT planar dose distribution was exported with a larger matrix size of 0.76 cm×0.76 cm cm so as to match the detector spacing of MatriXX. Subsequent to measurement on MatriXX in dose integration mode, both the TPS calculated and measured dose matrices were rescaled at 0.1 cm resolution using interpolation software provided in OmniPro IMRT analyzing software (V1.6). The calculated and measured dose distribution was then compared separately using gamma index analysis in OmniProIMRT software. Same gamma index acceptable criteria of 3%/3 mm selected for portal dosimetry, were used for the evaluation. Three gamma values, $\gamma_{\max},\gamma_{\mathrm{avg}}$ and $\gamma_{\%}\leq 1$ from both techniques of portal dosimetry and independent 2D ion chamber measurement were compared for each fields/subfields.

The performance of portal dosimetry was also tested for another five IMRT plans (45 fields) at different SDD ranging from 100 cm to 140 cm in steps of 10 cm at gantry zero. PDP algorithm predicted photon fluence/dose distribution at these extended SDDs was compared with the corresponding EPID measured fluence. The gamma values of each field obtained from these measurements were again compared with the corresponding gammas estimated at standard SDD of 100 cm. The effect of gantry rotation was also investigated for the same 45 IMRT fields by comparing gamma values estimated from measurement employing same clinically planned gantry angles with corresponding gamma values estimated from measurement at gantry zero and SDD of 100 cm. Differences in gamma values estimated from $P_{\text{PD}}$ and $P_{\text{MatriXX}}$ for each plan were statistically analyzed using Wilcoxon signed‐rank test and paired t‐test. Value of p≤0.05 was considered significant.

## III. RESULTS

Figure 1 represents the comparison of a) PDP predicted and EPID measured b) TPS calculated and MatriXX measured photon fluence/dose distribution for one of the representative IMRT field. Qualitative analysis of a line profile shows good agreement in both methods. The number of pixels falling beyond our set criteria seems slightly more in portal dosimetry, as represented by the red spots in Fig. 1(a). The mean value and standard deviation (SD) of $\gamma_{\max},\gamma_{\mathrm{avg}}$ and $\gamma_{\%}\leq 1$ estimated from all fields/subfields of each IMRT plan, and the overall mean (SD) of all 181 fields/subfields resulting from the 14 plans using portal dosimetry ($P_{\text{PD}}$) and MatriXX ion chamber ($P_{\text{MatriXX}}$) are depicted in Table 1. For all 181 fields/subfields, PDP predicted and EPID measured photon fluence/dose distribution agrees well with mean ± SD value for $\gamma_{\max}$=$2.02\pm 0.66,\;\gamma_{\mathrm{avg}}$ = 0.24±0.04, and $\gamma_{\%}1=99.43\%\pm 0.68\%$, respectively. Independent verification of the planned dose fluence from the same IMRT fields using MatriXX ion chamber array also resulted in comparable values of $\gamma_{\mathrm{avg}}$ = 0.25±0.07 and $\gamma_{\%}\leq 1=99.80\%\pm 0.44\%$. However, in all plans $\gamma_{\max}$ was consistently lower in $P_{\text{MatriXX}}$ with an overall mean ± SD value of 1.35±0.37 as compared to 2.02±0.66 from PPD. Variation of $\gamma_{\max}$ and $\gamma_{\%}\leq 1$ estimated from $P_{\text{PD}}$ and $P_{\text{MatriXX}}$ were significant (p≤0.05) in 12 (86%) and 8 (57%) plans, respectively. However, variation in $\gamma_{\mathrm{avg}}$ was significant only in two (14%) plans. In $P_{\text{PD}},\;\gamma_{\max}$ was found to be lesser for IMRT plans with 15 MV X‐rays, as compared to 6 MV.

**Table 1:** Mean and standard deviation (SD) of agreement of gamma parameters for each plan and all fields estimated using portal dosimetry $\left(P_{PD}\right)$ and independent method of MatriXX measurement $\left(P_{MatriXX}\right)$.

*Patient No.*	*Site*	*No of fields/subfields*	*Energy*	$P_{PD}$			$P_{MatriXX}$		
				$\gamma_{max}$	$\gamma_{avg}$	$\gamma_{\%}\leq 1$	$\gamma_{max}$	$\gamma_{avg}$	$\gamma_{\%}\leq 1$
1	H&N	7F/14SF	6 MV	1.95 (0.49)	0.26 (0.03)	99.24 (0.72)	1.58 (0.28)	0.26 (0.08)	99.61 (0.51)
2	H&N	9F/18SF	6 MV	2.31 (0.59)	0.26 (0.03)	99.48 (0.45)	1.37 (0.33)	0.29 (0.06)	99.83 (0.28)
3	H&N	7F/14SF	6 MV	2.06 (0.39)	0.26 (0.05)	98.74 (1.36)	1.34 (0.32)	0.26 (0.10)	99.84 (0.18)
4	H&N	7F/13SF	6 MV	1.80 (0.42)	0.26 (0.04)	99.22 (0.80)	1.24 (0.31)	0.27 (0.11)	99.59 (1.0)
5	Brain	7F/7F	6 MV	2.25 (0.78)	0.24 (0.02)	99.57 (0.23)	1.61 (0.22)	0.24 (0.07)	99.73 (0.28)
6	Pelvis	7F/7F	6 MV	2.66 (0.78)	0.26 (0.03)	99.07 (0.55)	1.83 (0.23)	0.30 (0.12)	99.51 (0.38)
7	Pelvis	7F/14SF	6 MV	2.84 (1.13)	0.26 (0.03)	98.92 (0.76)	1.79 (0.29)	0.29 (0.07)	99.59 (0.40)
8	Pelvis	7F/14SF	6 MV	1.99 (0.48)	0.25 (0.03)	99.59 (0.36)	1.19 (0.38)	0.24 (0.07)	99.87 (0.34)
9	Pelvis	7F/14SF	15 MV	1.79 (0.35)	0.22 (0.03)	99.71 (0.27)	1.58 (0.28)	0.26 (0.08)	99.61 (0.51)
10	Pelvis	7F/14SF	15 MV	1.68 (0.50)	0.21 (0.03)	99.80 (0.20)	1.37 (0.33)	0.29 (0.06)	99.83 (0.28)
11	Pelvis	7F/10SF	15 MV	1.50 (0.42)	0.23 (0.02)	99.90 (0.05)	1.34 (0.32)	0.26 (0.10)	99.84 (0.18)
12	Pelvis	7F/14SF	15 MV	1.88 (0.66)	0.20 (0.02)	99.82 (0.19)	1.24 (0.31)	0.27 (0.11)	99.59 (1.0)
13	Pelvis	7F/14SF	15 MV	1.79 (0.44)	0.22 (0.03)	99.50 (0.58)	1.61 (0.22)	0.24 (0.07)	99.73 (0.28)
14	Pelvis	7F/14SF	15 MV	1.90 (0.46)	0.22 (0.05)	99.39 (0.53)	1.83 (0.23)	0.30 (0.12)	99.51 (0.38)
All Patients			Mean	2.02	0.24	99.43	1.35	0.25	99.80
			SD	0.66	0.04	0.68	0.37	0.07	0.44

**Figure 1(a):**
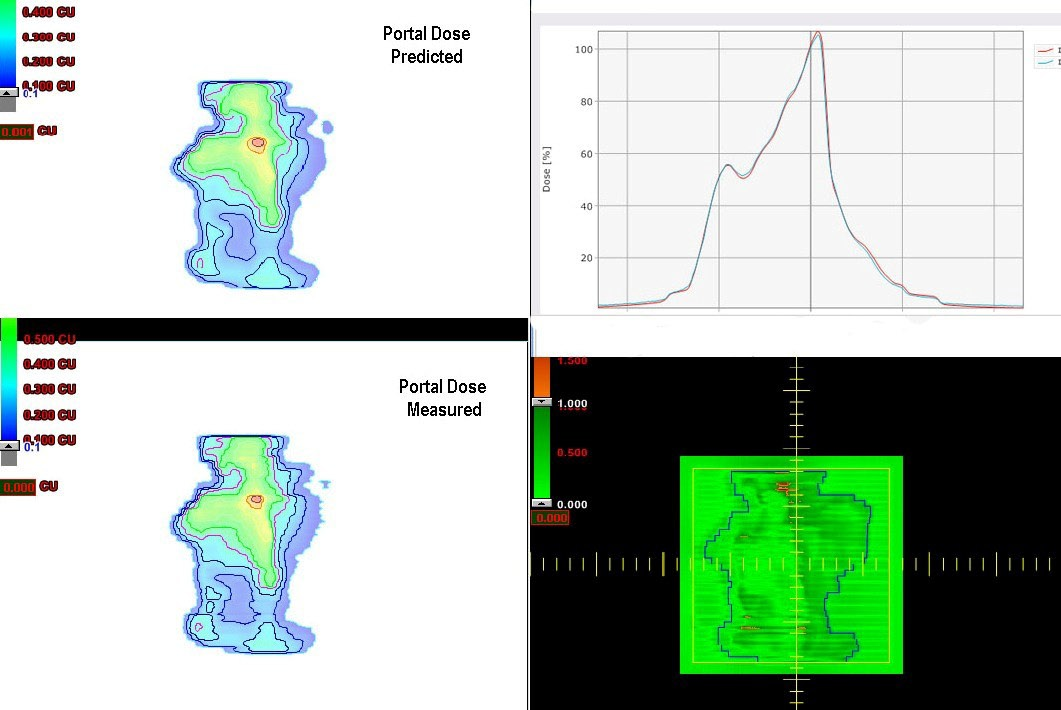
Comparison of PDP calculated and EPID measured planar dose distribution showing gamma analysis results (bottom right) and line profile agreement (top right).

**Figure 1(b):**
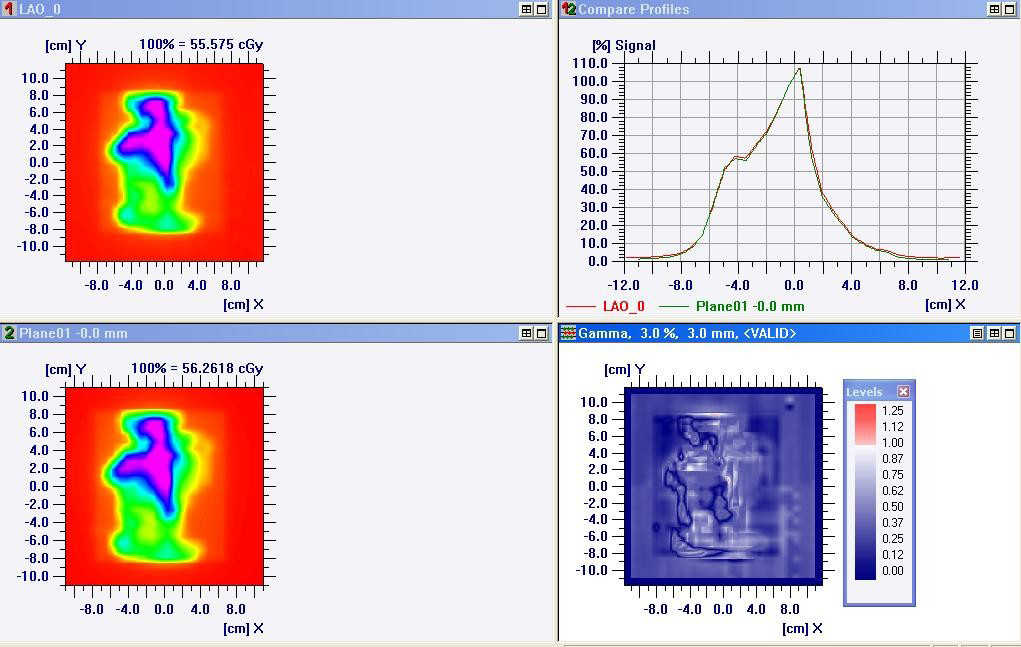
Comparison of TPS calculated and MatriXX measured planar dose distribution showing gamma analysis results (bottom right) and line profile agreement (top right).

**Figure 2:**
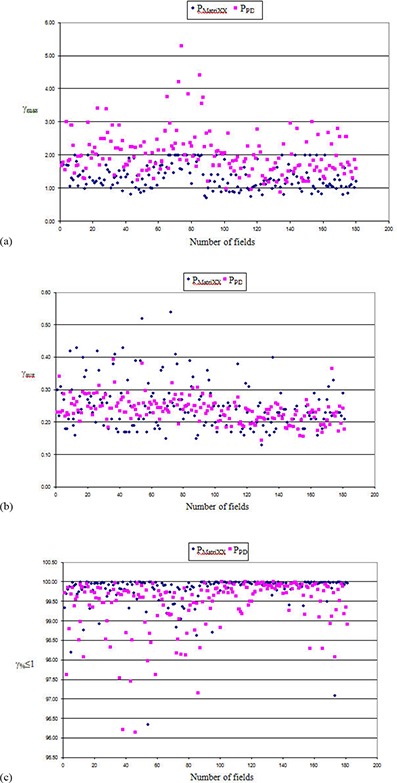
Composite scatter plot of $\gamma_{\max}$ (a), $\gamma_{\mathrm{avg}}$ (b) and $\gamma_{\%}\leq 1$ (c) for each of the 181 fields/subfields estimated from portal dosimetry ($P_{\text{PD}}$) and MatriXX ($P_{\text{MatriXX}}$) measurement.

Figure 2 shows the composite scatter plot of $\gamma_{\max}$ (Fig. 2(a)), $\gamma_{\mathrm{avg}}$ (Fig. 2(b)) and $\gamma_{\%}\leq 1$ (Fig. 2(c)) for each of the 181 fields/subfields estimated from $P_{\text{PD}}$ and $P_{\text{MatriXX}}$ measurement.

In $P_{\text{PD}}$, 23 (12.7%) fields/subfields showed $\gamma_{\max}$ value more than one SD from the mean (i.e, $\gamma_{\max}$>2.68) while 9 (5%) fields/subfields showed $\gamma_{\max}$ > 3.34 (mean ± 2 SD) with highest value at 5.3 in 1 (<1%) field. The number of fields/subfields with more than one (0.04) and two (0.08) SD from the mean (0.24) value of $\gamma_{\mathrm{avg}}$ were 20 (11%) and 3 (1.66%), respectively. The highest value of $\gamma_{\mathrm{avg}}$ from portal dosimetry was 0.40. The number of fields/subfields in $P_{\text{MatriXX}}$ which shows $\gamma_{\max}$ and $\gamma_{\mathrm{avg}}$ values larger than one SD from the mean (1.72 and 0.32) were 35 (19.30%) and 25 (13.81%), respectively. All fields/subfields had $\gamma_{\max}<2.09(\;\text{mean}\pm 2\text{SD})$; however, in 10 (5.5%) fields/subfields, $\gamma_{\mathrm{avg}}$ values were more than 2 SD from the mean (0.39) attaining highest at 0.54 in one field. In both the methods, almost all fields showed more than 98% of all pixels in the fields having gamma value at least 1 ($\gamma_{\%}\leq 1$) except for 8 fields (4.4%) in $P_{\text{PD}}$ and 2 (1.2%) in $P_{\text{MatriXX}}$ where gamma value was less than 98% but better than 96%.

**Figure 3:**
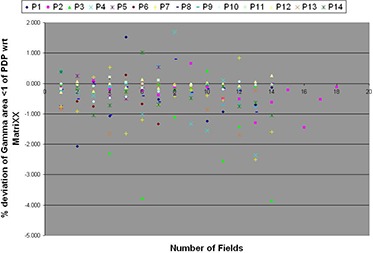
Percentage deviation of $\gamma_{\%}\leq 1$ values of portal dosimetry as compared to MatriXX measurement for each of the individual 181 fields. P1, P2 ‥ P14 represent IMRT plans.

Of the 14 IMRT plans, one plan in $P_{\text{PD}}$ had 5 out of 14 fields (35.71%) showing $\gamma_{\max}>2\;\text{SD}$ from the mean. Qualitative analysis of these portal images showed maximum gamma values located in the periphery of the large IMRT fields. Though a similar pattern was observed in $P_{\text{MatriXX}}$ for the same plan, values of $\gamma_{\max}$ estimated from $P_{\text{MatriXX}}$ were less than 2 SD. On the other hand, the number of fields having $\gamma_{\mathrm{avg}}>2\;\text{SD}$ from mean was more in $P_{\text{MatriXX}}$. Two fields (<15%) each of the three IMRT plans in $P_{\text{MatriXX}}$ showed $\gamma_{\mathrm{avg}}>2\;\text{SD}$ from the mean. The percentage deviation of $\gamma_{\%}\leq 1$ between portal dosimetry and MatriXX for each of the individual 181 fields/subfields from 14 IMRT plans is shown in Fig. 3. The number of fields/subfields with more than 2% and 3% deviations in $\gamma_{\%}\leq 1$ values were seven (3.8%) and two (1.1%) fields, respectively. The maximum deviation observed in any of the single field/subfield was ‐3.85%.

Table 2 represents the mean ± SD of gamma difference estimated from field by field comparison of gamma values at different SDD with respect to that at 100 cm SDD. The mean ± SD of gamma difference between portal images acquired at clinically planned gantry angle and corresponding portal images acquired at gantry zero for all 45 fields/subfield from five IMRT plans are also presented in Table 2. In comparison to gamma values at 100 cm SDD, all three gammas have shown overall improvement at different extended SDD. The $\gamma_{\max}$ and $\gamma_{\mathrm{avg}}$ values were found to decrease with a mean ± SD of 0.16±0.20 and 0.03±0.03 at 110 cm SDD to 0.35±0.45 and 0.05±0.04 at 140 cm SDD, while $\gamma_{\%}\leq 1$ increases marginally to 0.26±0.58 at 110 cm SDD and 0.29±0.47 at 140 cm SDD. The effect of gantry rotation on portal dosimetry outcome was almost negligible indicated by $\gamma_{\max},\gamma_{\mathrm{avg}}$ and $\gamma_{\%}\leq 1$ values at clinically planned gantry angles being different from corresponding values at gantry zero with a mean of 0.06, 0.01 and ‐0.29, respectively.

**Table 2:** Mean ± SD of field‐by‐field differences in gamma values at a) different SDD as compared to standard 100 cm SDD with gantry zero, and b) clinically‐planned gantry angles as compared to gantry zero and 100 cm SDD.

*No of Fields = 45*	*SDD*	$\gamma_{max}$	$\gamma_{avg}$	$\gamma_{max}$
	110	−0.16±0.20	−0.03±0.03	0.26±0.58
	120	−0.22±0.31	−0.03±0.03	0.26±0.64
	130	−0.35±0.37	−0.05±0.04	0.27±0.59
	140	−0.35±0.45	−0.05±0.04	0.29±0.47
	Diff Gantry & SDD=100 cm	0.06±0.14	0.01±0.02	−0.29±0.44

SDD = source‐to‐detector distance

## IV. DISCUSSION

In our Institute, pretreatment verification of IMRT plans have been carried out using the MatriXX 2D ionization chamber array and cross verification by 0.65 cc ionization chamber absolute point dose measurement, in case any discrepancies are found beyond our acceptable limit. Routinely we evaluate the agreement between TPS calculated and MatriXX measured planar dose distributions and absolute dose as an integration of entire fields in the plan – not as individual fields as has been done in the present study. Any plan which deviates from our acceptance criteria of $\gamma_{avg}<0.5,\;\gamma_{\%}\leq 1$ better than 95% as well as more than 2% variation in absolute dose would be investigated further. While the MatriXX 2D ion chamber array has the advantage of measuring both relative and absolute dose of a plan in a single setting, a possible drawback of MatriXX could be the limited resolution of 0.76 cm as compared to 0.039 cm of aSi1000. Low et al.^(^ ^25^ ^)^ reported that the large active volume of ionization chambers exhibit volume averaging effect in steep dose gradient regions. A volume‐averaging effect corresponding to full width at half maximum (FWHM) of about 0.58 cm was reported due to the finite size of MatriXX.^(^ ^26^ ^)^ The multiple acquisition (MA) sequence proposed by Spezi et al.^(^ ^27^ ^)^ to increase the resolution of dose measurement point was not feasible for the present study due to the large number of individual measurements required for split and non‐split fields. In our study, in order to partially overcome the probable limited resolution of MatriXX, the TPS calculated IMRT planar dose distribution was exported with a larger matrix size of 0.76 cm×0.76 cm so as to match the detector spacing of MatriXX. Subsequent to measurement on MatriXX, both TPS calculated and measured dose matrices were rescaled at 0.1 cm resolution using interpolation software provided in OmniPro IMRT analyzing software. Our results from $P_{\text{MatriXX}}$ measurement were satisfactory for all IMRT plans and fields and were within the acceptable criteria of $\gamma_{avg}<0.5$,$\gamma_{\%}\leq 1$ better than 95%. The similarity of $\gamma_{\mathrm{avg}}$ and $\gamma_{\%}$ values in both MatriXX and portal dosimetry also suggest that the use of system interpolation software can overcome the limited resolution of MatriXX 2D array. Very limited data on pretreatment verification of patient‐specific IMRT plan using MatriXX are available for comparison in the literature. Stasi et al.^(^ ^8^ ^)^ in their four‐patient (19 fields) study, showed $\gamma_{\%}\leq 1$ value of 97.7%±0.5% for MatriXX when compared to film dosimetry and 97.6%±0.5% for MatriXX versus TPS. Our $\gamma_{\%}\leq 1$ values were better than 99% in all 18 IMRT plans (181 fields), with an overall mean (SD) of 99.8%±0.44%. Other gamma parameters were not available for comparison.

Portal dosimetry as a quality assurance measure for pretreatment verification of IMRT plans has been reported from a few centers using mostly in‐house developed software.^(^ ^18^ ^–^ ^21^ ^)^ One of the reasons for limiting the vast application of portal dosimetry in the clinic setting could be due to the lack of commercially‐available software required for EPID dosimetry. Varian (Varian Medical System, Palo Alto, CA) introduced a portal dose prediction algorithm in Eclipse TPS based on the study by Van Esch et al.^(^ ^18^ ^)^ We have reported portal dosimetry results for various clinical sites using commercially available portal dose prediction algorithm in Eclipse TPS. Our results shows very good agreement between predicted and measured dose distribution in all plans with overall mean ± SD gamma values: $\gamma_{\max}=2.02\pm 0.66,\;\gamma_{avg}=2.24\pm 0.04$ and $\gamma_{\%}\leq 1=99.43\%\pm 0.68\%$, respectively. McDermott et al.^(^ ^19^ ^)^ in their study of 20 prostate plans (five fields/plan) using in‐house developed EPID dosimetry software reported overall mean $\gamma_{\max}=2.52$,$\gamma_{avg}=0.39$ and $\gamma_{\%}\leq 1=98.7\%$. The in‐house developed back‐projection algorithm was based on reconstruction of aSi EPID measured transit image to phantom mid‐plane dose distribution. In another large series of 75 patients (316 fields) treated with dynamic IMRT in various sites, VanZijtveld et al.^(^ ^20^ ^)^ reported mean $\pm SD\;\gamma_{avg}=0.43\pm 0.13$ and $\pm SD\;\gamma_{avg}=0.43\pm 0.13$, respectively. The investigators have used fluoroscopic camera based EPID and in‐house portal dose prediction software implemented in commercially‐available CadPlan TPS (Varian Medical Systems, Palo Alto, CA). Recently, portal dosimetry results employing commercially available aSi1000 and PD prediction software from Varian was reported from Howell et al.^(^ ^21^ ^)^ In this largest series of 152 IMRT plans (1152 treatment fields) of various sites, the authors reported overall mean $\pm SD\;\gamma_{max}=2.4\pm 0.8,\;\;\gamma_{avg}=0.33\pm 0.13$ and $\gamma_{\%}>1=4.1\%\pm 6.2\%$. They also reported higher gamma values for more complex target geometry (e.g., H&N), split fields and simultaneously integrated boost (SIB) as compared to simple target, non‐split fields and non‐SIB. Our findings are also in agreement with this study. The majority of the IMRT plans chosen in our study were complex H&N, prostate with nodes, and cervix with nodes involving split fields in 81% of the fields which were also planned for simultaneously integrated boost (SIB) dose in many cases. The overall as well as site‐specific gamma values were slightly better in our study. Recently, Lee et al.^(^ ^28^ ^)^ proposed a simple algorithm independent of treatment planning system. EPID images of 14 clinical IMRT fields from two different sites were acquired and converted into planar dose map using the authors' in‐house developed algorithm and compared directly with the ADAC Pinnacle TPS calculated dose map. Gamma analysis of the EPID, film, and Pinnacle planar dose maps generated for the clinical IMRT fields showed $\gamma_{\%}\leq 1$ of approximately 97%. The variations observed in different studies including ours could be attributed to different detector technology and portal image to dose conversion software, as well as the IMRT plans used in the studies.

Ideally, pretreatment verification of patient‐specific IMRT plan is carried out using a QA system independent of TPS. However, portal dosimetry solution used in this study employs EPID system, portal dose prediction algorithm, portal image acquisition and postprocessing algorithm all from the same vendor (Varian Medical Systems). Therefore, the performance of this new portal dosimetry system was independently validated using MatriXX 2D ion chamber array before its use clinically. Moreover, while gamma analysis data from clinical IMRT plan measured using MatriXX and portal dosimetry are available seperately, to our knowledge, no data are available comparing MatriXX results with that from portal dosimetry in a large number of clinically planned IMRT fields. In our study, we have compared the dosimetric outcome from these two techniques covering complicated and most common clinical sites treated with IMRT. Direct comparison of EPID and MatriXX measured fluence maps was not done as they function differently. The gamma values estimated from both techniques were identical, except for $\gamma_{\max}$ (as shown in Fig. 2(a). The comparatively few number of fields showing less deviation in $\gamma_{\max}$ value in $P_{\text{MatriXX}}$ as compared to $P_{\text{PD}}$ could be due to software interpolation of lesser measurement values used in $P_{\text{MatriXX}}$. While the differences in gamma values estimated from MatriXX and portal dosimetry were significant in some of the plans, the values were well within the acceptable limit.

## V. CONCLUSIONS

The overall results of patient‐specific IMRT fluence verification using portal dosimetry and MatriXX 2D ion chamber array is comparable except for $\gamma_{\max}$, which is higher in portal dosimetry. However, all parameters are well within the clinically acceptable values. Portal dosimetry outcomes were independent of gantry rotation, and gamma values improve with increase in source‐to‐detector distance. Based on the result of range of the fields studied, portal dosimetry can be consider as an alternative method of 2D detector array or vice versa for pretreatment verification IMRT plan.
